# Bare Neck and Arms

**Published:** 1861-02

**Authors:** 


**BARE NECK AND ARMS.**—There is, perhaps, not an eminent physician in any system of practice, who will not declare, with a distinguished medical practitioner, now deceased: "I believe, that during the twenty-six years I have followed my profession in this city, twenty thousand children have been carried to the cemeteries, a sacrifice to the absurd custom of exposing their arms naked."

